# Reliability and validity of the Turkish version of the Physical Activity Questionnaire for Older Children (PAQ-C)

**DOI:** 10.3906/sag-1806-212

**Published:** 2019-02-11

**Authors:** Leyla ERDİM, Ayşe ERGÜN, Sema KUĞUOĞLU

**Affiliations:** 1 Department of Midwifery, Faculty of Health Science, İstanbul University-Cerrahpaşa, İstanbul Turkey; 2 Department of Public Health Nursing, Faculty of Health Science, Marmara University, İstanbul Turkey; 3 Division of Nursing, Faculty of Health Science, İstanbul Medipol University, İstanbul Turkey

**Keywords:** Child, exercise, psychometrics, schools, students, surveys and questionnaires

## Abstract

**Background/aim:**

The Physical Activity Questionnaire for Older Children (PAQ-C) is one of the most frequently used questionnaires for evaluating moderate to vigorous levels of physical activity in children. However, a Turkish version of the questionnaire was not available. This study aimed to create a Turkish version of the PAQ-C and examine its psychometric characteristics and factor structure.

**Materials and methods:**

The study was conducted with 784 primary school students (ages 9–14 years). The PAQ-C was examined for content and construct validity and analyzed in terms of reliability. Content validity was evaluated with the content validity index (CVI), construct validity, exploratory factor analysis (EFA) (n = 388), and confirmatory factor analysis (CFA) (n = 396). For reliability analysis, Cronbach’s alpha and the intraclass correlation coefficient (ICC) were calculated. The factor construct was assessed with corrected item total correlations (CITCs).

**Results:**

The CVI for the PAQ-C was 0.95. EFA revealed a two-factor construct of ‘at school’ and ‘extracurricular’. CFA confirmed the two-factor construct. Factor loadings varied between 0.41 and 0.80. Cronbach’s alpha was 0.77 and ICC was 0.91 for the test-retest for the entire sample. All CITCs were >0.20.

**Conclusion:**

The PAQ-C is a valid and reliable instrument that can be used for Turkish children.

## 1. Introduction

Children and young people today are growing increasingly more inactive. Children are walking or riding bicycles less, and cars are the most popular vehicles of transportation. The US Human Health Services Department recommends daily physical activity (PA) of at least 60 min or more for children and adolescents ages 6–17 (1). The results of the US Youth Risk Behavior Surveillance Survey conducted in 2015 with students in grades 9–12, however, showed that in the last 7 days before the survey, only 27.1% had engaged in at least 60 min of PA that would raise their heart rates (2). In Turkey, Erginöz et al. (3) reported in a study that examined the level of PA of 5552 Turkish schoolchildren, ages 11–15, that only 20% had participated in an adequate amount of PA. 

The increases in insufficient PA levels and in rates of obesity point to the importance of determining the factors that affect the PA level of children and young people and stress the need for programs to increase PA rates. Valid and reliable instruments are needed in order to make an effective measurement of research results on PA. Objective and subjective measurement methods are utilized to assess the PA levels of children and adolescents (4). It is difficult to use objective measurement methods (e.g., double-labeled water, heart rate monitors) in large population groups such as schools (4,5). The Physical Activity Questionnaire for Older Children (PAQ-C) is one of the self-reporting scales used in such measurements (6). The use of self-reporting scales is easy and economical for large samples. This feature makes the PAQ-C an attractive method to use. At the same time, the attractiveness of the PAQ-C also lies in the fact that the items contained in it remind children to engage in PA (6). The PAQ-C, developed in Canada for the purpose of assessing the moderate-vigorous physical activity (MVPA) levels of children of an average age of 8–14 in grades 4–8, is a 7-day recall instrument based on self-reporting. The objective of the PAQ-C is to determine the general PA levels of children at school (7,8). The scale was tested for validity and reliability with Canadian children and it was found to be acceptable in terms of its item and scale properties, test and retest reliability, internal consistency, sensitivity to gender differences, and construct and convergent validity (7,8). The questionnaire was also tested for validity and reliability in the USA (9,10), Iran (11), the Netherlands (5), the UK (12), China (13), and Spain (14). The factor construct of the PAQ-C showed dissimilarities in different cultures. Janz et al. (10) defined a single-factor construct for their sample of American children while Moore et al. (9) identified a two-factor construct in their study with Hispanic children. Wang et al. (13) identified a single-factor construct in the scale for Chinese children while Thomas and Upton (12) discovered a two-factor construct for British children. 

Testing the PAQ-C for intercultural validity will be useful for assessing the PA level of children speaking the Turkish language who live in different countries around the world. At the same time, this will provide an opportunity for making comparisons between countries. 

This study aimed to create a Turkish version of the PAQ-C and examine its psychometric characteristics and factor structure. 

## 2. Materials and methods

### 2.1. Study design and sample

This methodological study was conducted with students enrolled in grades 4–8 (ages 9–14) of a public primary school in northwest Turkey, in the district of Fatih, İstanbul. The literature on scale adaptation recommends that the size of a sample equal 10–20 times the number of items. It is also recommended that samples for studies in which confirmatory factor analysis (CFA) will be performed be conducted with 300–500 individuals (15). The universe of the study consisted of the students enrolled in the 46 primary schools located in the district of Fatih. To select the sample, the primary schools in the district were listed via an electronic medium and 9 of these were randomly chosen. Lots were cast to select one class from each grade (grades 4–8) to create the sample. 

A total of 981 students with no disabilities and whose parents agreed to have their children participate in the research were recruited into the study. Seventy-seven students with incomplete questionnaires and 120 students who declared that they had been sick during the previous week were excluded from the study prior to the analysis. The study was ultimately conducted with the data of 784 students. 

To test the test-retest reliability of the questionnaire, one school was selected by a casting of lots among the schools taken into the sample. From the students in grades 4, 5, 6, 7, and 8 who had been administered the questionnaire, 114 (girls = 58, boys = 56) were selected to be retested 7–10 days after the first test. 

### 2.2. The questionnaire (PAQ-C)

From the 10 items constituting the scale, 9 of the items are used to calculate activity scores. The 10th item assesses whether or not the child engaged in normal activity in the previous week despite being sick or having some other interference. This item, however, is not included in the calculation of the activity score. The first question on the PAQ-C is in the form of an activity checklist specifying 22 common leisure and sport activities and another category of ‘Other’. The responses to this question are evaluated on the basis of a 5-point rating (1 = no activity at all, 5 = 7 times or more), from which a mean score is calculated; higher points indicate a greater extent of PA. The clear and specific mention of the 22 activities in this questionnaire provides the advantage of a reminder to respondents. The remaining 8 questions relate to an evaluation of activities performed during the day or in specific intervals of time during the week (e.g., physical education class, recess, noontime, after school, in the evening, over the weekend). These items are scored on the 5-point scale and higher scores indicate a higher level of activity. The overall PAQ-C score is obtained by adding the scores of items 1–9 and the final PQ-C activity summary score is the mean of the scores of these 9 items. While a mean score of 1 indicates a low level of PA, a mean score of 5 signifies a high level of PA (6). 

### 2.3. Translation of the questionnaire and pilot testing

In order to be able to use the PAQ-C in the study, permission was obtained from Kent C. Kowalski via email. Kent C. Kowalski emailed the data related to the questionnaire and its implementation. The original questionnaire is in English. Three independent individuals fluent in both languages translated the questionnaire into Turkish from the original so that it could be evaluated for language equivalence and cultural relevance. The statements in the Turkish version were reviewed and compared with the original questionnaire. The most suitable versions of the statements were chosen for best comprehension and a single Turkish questionnaire was created. Two different linguists independent of the initial translators executed the back translations of this new Turkish version. Back translation is very important in the verification of the translation of questionnaires. The process consists of having an instrument translated into the desired tongue and then enlisting another translator to translate the version created back into the original language (16). The original English questionnaire and its back-translated English version were compared and it was observed that there were no differences in meaning. 

The Turkish and the English form of the instruments were sent out to an expert panel consisting of 13 university faculty members, including two pediatricians, a public health nurse, nine pediatric health nurses and a physiotherapist with backgrounds similar to those of the translators, for an evaluation of the suitability of the Turkish version in terms of wording and cultural concepts. The experts were asked to evaluate the items in the instruments on the basis of the content validity index (CVI), such that 1 = unsatisfactory and 4 = very satisfactory. For the content to be 80% satisfactory in terms of validity, the experts had to give each item of the instruments a 3- or 4-point score (17). 

During the cultural adaptation, in line with the common view of the experts, the activities among the 22 different physical activities in the first question on the PAQ-C that were typically not performed in Turkey (baseball/softball, American football, badminton, street hockey, field hockey, cross-country, and ice hockey) were removed. After some small revisions were made in the questionnaire on the basis of the feedback from the experts, 15 different activities were left in the selection of PA. 

A pilot test was implemented to understand whether or not the children were able to comprehend the items on the questionnaire. The questionnaire was applied to 10–15 random participants from each class (grades 4–8), meaning a total of 84 students. During the pilot testing of the scale, the ‘aerobics’ choice in the first question of the questionnaire was also removed because the children were unable to understand it. In the final version, the first question on the Turkish PAQ-C consisted of 14 choices of activity. It was seen that the students were able to comprehend the other questions in the questionnaire and therefore the Turkish version was given its final form. The students participating in the pilot run and the pilot data were not included in the study sample. The Turkish and the English forms of the Physical Activity Questionnaire for Older Children are presented in the Appendix.

### 2.4. Procedures

Before the study, the University Ethics Committee approved all procedures. In addition, the informed written consent of a parent or legal guardian was obtained, along with the assent of the participants, before data collection. The questionnaire was distributed in the classrooms in the morning hours on routine schooldays, Monday to Friday, and then collected from the students. The number of students in each class varied between 20 and 25. The researchers obtained the verbal consent of the classroom teachers and students before administering the questionnaire. Then, after the students were provided with a short explanation about the purpose of the questionnaire, all of the questions were read out loud and any questions the students had were answered; no guidance was offered however in any way. The children were given an average of 20 min in which to fill out the questionnaire.

To test the test-retest reliability, the questionnaire was administered a second time to 114 students 7–10 days later. The retest analysis was performed with 71 completed questionnaires.

### 2.5. Statistical analysis

The data were analyzed using SPSS 21.0 (IBM Corp., New York, NY, USA). Features were defined with frequency distribution and using means and standard deviation (SD). Content validity and construct validity were examined to determine the reliability of the PAQ-C. Content validity was evaluated with the CVI, which is recommended for an assessment of expert ratings (15,17). Lynn (17) stated that the CVI must be at least 0.83. Cross-validation was applied to the exploratory (EFA) and confirmatory factor (CFA) analyses carried out for construct validity. The purpose of cross-validation is to observe if the model obtained from EFA can be repeated in a second sample (18). With this aim, the 784 students included in the study were randomly divided into two groups. EFA was carried out for the first group of the sample (n = 388) and CFA was executed for the second group (n = 396). The CFA was performed using LISREL 9.20 software. To test the agreement between the data and the CFA model, the chi-square goodness, GFI (goodness of fit index), AGFI (adjusted goodness of fit index), CFI (comparative fit index), RMSEA (root mean square error of approximation), SRMR (standardized root mean square residual), and NNFI (nonnormed fit index) fit indices were examined. The criteria for the fit indices were <0.10 for RMSEA, ≥0.90 for CFI, >0.80 for AGFI, >0.90 for GFI, and <0.10 for SRMR (19–23). 

Cronbach’s alpha coefficient of internal consistency or reliability was calculated (24). In terms of reliability, it is important that the Cronbach’s α coefficient of a scale be 0.70 or above (25). The intraclass correlation coefficient (ICC) was calculated to determine test-retest reliability and an ICC result of 0.80 was considered excellent (26). Item/total correlations were assessed with corrected item total correlations (CITCs). A CITC of more than 0.20 in an item signifies that the factor can serve its purpose to a significant degree (20,27). Significance levels were set at P < 0.05. 

## 3. Results

### 3.1. Descriptive statistics 

Of the students, 48.7% were girls (n = 382), 51.3% were boys (n = 402), and the mean age was 11.00 ± 1.34 years. Table 1 presents some descriptive statistics of the PAQ-C scores of the boys, girls, and overall sample. The PAQ-C total score for the overall sample was 3.6 (SD = 0.73). The male students’ PAQ-C scores were statistically and significantly higher than the girls’ scores (t = –4.50, P > 0.001). The score of the 9–11 age group (28.75 ± 6.48) was significantly higher than the 12–14 age group (27.66 ± 6.75) (t = 2.10, P = 0.036). 

**Table 1:** Descriptive statistics.

Items	Girls (n = 382)		Boys (n = 402)		Overall (n = 784)		Mean	SD	Mean	SD	Mean	SD
	1. Spare time activity checklist	2.03	0.55	2.20	0.62	2.11	0.59
2. Physical education	4.52	1.00	4.51	0.99	4.52	0.99
3. Recess	3.07	1.44	3.56	1.52	3.33	1.50
4. Lunch	2.91	1.43	3.24	1.56	3.08	1.51
5. After School	3.16	1.30	3.32	1.39	3.24	1.35
6. Evenings	2.67	1.22	2.81	1.37	2.74	1.30
7. Weekends	3.23	1.21	3.45	1.28	3.35	1.25
8. Describes you best	2.78	1.26	3.11	1.35	2.96	1.32
9. Activity frequency for each day of the last week	2.96	0.90	3.24	1.00	3.11	0.96
PAQ-C	3.04	0.69	3.27	0.74	3.16	0.73

### 3.2. Corrected item total correlations, internal reliability, and test-retest reliability 

Cronbach’s α for the overall sample was found to be 0.77 (girls α = 0.76; boys α = 0.77). Table 2 shows the CTICs and the factor loadings for the first (n = 388) and second (n = 396) samples. The CITCs were found to be between 0.25 and 0.69. In the ICC analysis, the second application (n = 71) of the scale was carried out 10 days after the first application and the ICC for the overall sample was found to be 0.91 (Table 2). 

**Table 2:** Corrected item total correlations (n = 784) and factor loadings for sample 1 (n = 388) and sample 2 (n = 396).

	CITCs	EFA (n = 388)		CFA (n = 396)	
Item		Factor 1	Factor 2	Factor 1	Factor 2
1. Spare time activity checklist	0.44	0.61		0.55	
2. Physical education	0.25		0.68		0.41
3. Recess	0.38		0.62		0.72
4. Lunch	0.34		0.71		0.50
5. After school	0.53	0.71		0.68	
6. Evenings	0.47	0.67		0.59	
7. Weekends	0.56	0.73		0.68	
8. Describes you best	0.57
0.68		0.67	
9. Activity frequency for each day of the last week	0.69	0.81		0.80	

CITCs: Corrected item total correlations; EFA: exploratory factor analysis; CFA: confirmatory factor analysis.

### 3.3. Content validity

The mean of the points the thirteen experts gave the items of the PAQ-C was found to be 3.82 (min 3, max 4) and the overall CVI was 0.95. 

### 3.4. Construct validity

To study the construct validity of the PAQ-C, the Kaiser–Meyer–Olkin measure of sampling (KMO test) and Bartlett’s sphericity test were performed to first assess whether the sample size in the EFA was suitable for factor analysis. In this study, we found the KMO measure of sample adequacy to be 0.86, and saw that the chi-square value for Bartlett’s sphericity test was 1698.421, df = 36 (P < 0.000). The results of the EFA showed that the scale’s eigenvalue was over 1, pointing to a two-factor construct. We loaded the six items (Q1, Q5, Q6, Q7, Q8, Q9) related to the extracurricular physical activities shown in the scale into factor 1 and factor loadings varied between 0.61 and 0.81. Factor 1 explained 38.48% of total variance. The three items (Q2, Q3, Q4) related to physical activities at school in the questionnaire were loaded into factor 2. Factor 2 explained 13.55% of total variance and factor loads were between 0.62 and 0.71 (Table 2). 

To confirm the two-factor construct of the PAQ-C obtained in the first sample, CFA was performed for the second sample. The chi-square (χ2) value, which varies according to sample size, was found to be 56.54 in the CFA, degrees of freedom (df) were 26, and χ2/df was 2.174. The fit indices showed the following results: RMSEA = 0.054, NNFI = 0.95, CFI = 0.97, GFI = 0.97, SRMR = 0.037, and AGFI = 0.95. Parameter estimates (standardized coefficients) ranged from 0.41 to 0.80 for all items in the model and the correlation coefficients among the factors was 0.45 (Figure).

**Figure 1:**
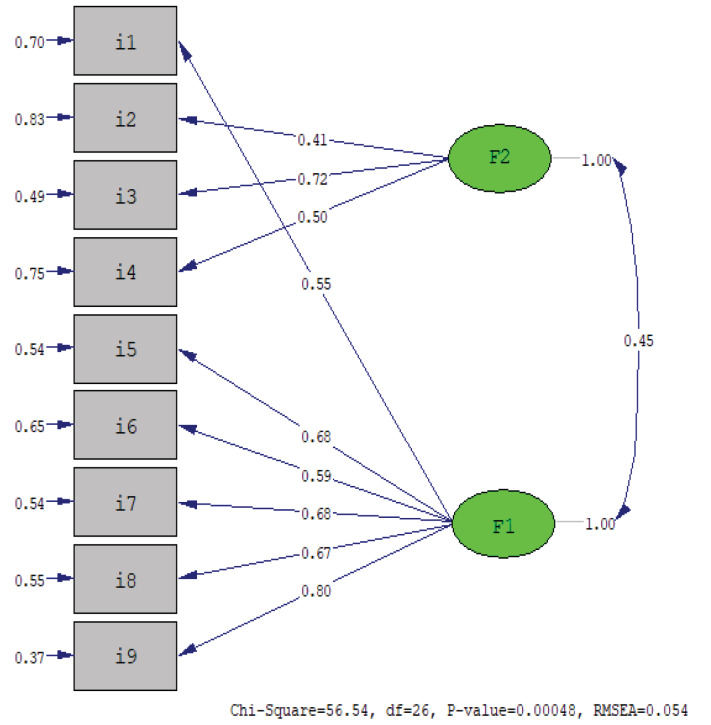
Factor structure model for PAQ-C (n = 396).

## 4. Discussion

The study results showed that the Turkish version of the PAQ-C that was implemented to assess the MVPA levels of children aged 9–14 was valid and reliable.

The mean of the activity checklist, which is the first item of the questionnaire (Q1), was found to be a little lower than those of the other items 2.11 ± 0.59, an outcome that is similar to the result reported (1.87 ± 0.59) in a study (12) conducted with British children. This was interpreted to be a consequence of the numerous activities queried on the checklist and due to the fact that the children did not perform most of them. Because of this, it might be suggested that limiting this item to activities that children most frequently engage in would facilitate another assessment. 

On the other hand, the mean of the second item on the questionnaire (Q2) related to the PA carried out during physical education classes was found to be high. This was similar to reports of British (4.14 ± 0.80) (12) and Chinese (4.04 ± 0.98) (13) samples. In Turkey, physical education classes are limited to 2 h a week (28–30) and for this reason it would be expected that children are active in this class. Their being active, however, may not show that they have a high level of activity in general. Most item means in the questionnaire were close to the center of the range of values.

The PA total scores of all of the students (3.16 ± 0.73) were observed to be close to the results of studies conducted with children of different races in similar age groups. For example, scores reported were 3.36 ± 0.80 for European American children, 3.37 ± 0.69 for African American children, and 3.19 ± 0.64 for Hispanic children (9). Scores of 3.49 ± 0.68 and 3.36 ± 0.67 were reported from British samples (12). Our results were significantly higher than study results with Chinese children (2.62 ± 0.68) (13). 

### 4.1. Reliability

It is important that the reliability coefficient of a scale be 0.70 or above (25). In the reliability analysis, it was seen that Cronbach’s α = 0.77 for the overall sample was at an acceptable level of internal consistency. The results of our study were similar to those reported in studies with Canadian (α = 0.79–0.89) (7), American (α = 0.72–0.76) (10), Hispanic (α = 0.76) (14), European American (α = 0.75) (9), and Chinese children (α = 0.79) (13). 

In the retest reliability analysis to assess the consistency of the scale over time, our results were high (ICC = 0.91), as in the results of studies with Hispanic (ICC = 0.96) (14) and Chinese (ICC = 0.82) (13) samples. This outcome demonstrated that the consistency of the scale over time for the target population was excellent (26). 

In the item analysis carried out to determine the differential predictive power of the overall score of the items on the PAQ-C (20,27), the CITCs were in the range of 0.25–0.69, the lower limit being over 0.20, similar to the results obtained from the Chinese sample (0.29–0.72) (13). These results showed that the item/scale relationship of the PAQ-C is acceptable for this population (20,27). 

### 4.2. Validity 

#### 4.2.1. Content validity

The agreement between the experts regarding the comprehensibility and suitability of the items of the scale is accepted as an indication of the scale’s content validity (17). It was observed that there was agreement among the 13 experts whose opinions were enlisted to assess the content validity of the PAQ-C, and CVI was 0.95. Therefore, it was concluded that the PAQ-C is culturally compatible and fulfills the criteria of content validity (17). 

#### 4.2.2. Construct validity

The data set of the nine-item PAQ-C were randomized and divided into two groups to test construct validity; EFA was performed for the first half and CFA for the second half. The EFA in the first sample produced a two-factor construct. Accordingly, the items referring to the ‘extracurricular’ physical activities in the questionnaire (Q1, Q5, Q6, Q7, Q8, Q9) were loaded into factor 1 and the physical activities carried out ‘at school’ (Q2, Q3, Q4) were loaded into factor 2. Two studies were found in the literature testing the validity of the PAQ-C with EFA and CFA (9,12). It was stated in the study by Moore et al. (9) that EFA results exhibited a three-factor construct. The authors removed Q4 and thereby obtained a construct model similar to that of the present study, producing a two-factor construct of ‘extracurricular’ and ‘at school’ (9). In another study with British students, CFA results obtained after EFA pointed to a two-factor construct, consistent with our own study (12). In the present study, without having to retest the model, ‘extracurricular’ physical activities were loaded into factor 1 and physical activities performed ‘at school’ were loaded into factor 2. 

The CFA applied to the second sample in the study supported the two-factor construct obtained from the EFA from the first sample. All of the other goodness of fit statistics, excluding chi-square, were at the level desired; the factor loadings of all items of the questionnaire were found to be higher than 0.30 (29). This outcome, as in the results of similar studies, showed that the scale had a two-factor structure that measured the level of MVPA over the last 7 days (9,12). Furthermore, our study also demonstrated that, similar to the results of studies carried out with Hispanic (9) and British (12) children, the factorial structure of the PAQ-C was sensitive to the setting in which PA was performed and factors could be defined as ‘extracurricular’ and ‘at school’ (9,12). That PAQ-C can differentiate between settings where activity takes place is an advantage for school-based programs and policies. The scale makes it possible to assess the aspects in which children’s PA is inadequate and therefore provides the groundwork for new programs that may be implemented.

The study had various limitations. The first of these was the use of the PAQ-C based on only self-reporting in our investigation of validity and the fact that no device was used to measure activity. This limitation may have prevented a more accurate interpretation of our actual results. At the same time, the PAQ-C developed for children of ages 8–14 was employed for children ranging in age from 9 to 14 and this may be considered a second limitation. 

In conclusion, this study was the first to explore the construct validity and reliability of the PAQ-C among Turkish children and the results demonstrated that the questionnaire is a valid and reliable instrument that can be employed in evaluating the MVPA levels of Turkish children. Since the questionnaire is easy to use and apply, can be implemented at low cost, and can be filled out very quickly, it may be used in larger-scale PA research. It will also serve as a tool for evaluating PA among Turkish children living abroad, who can benefit from being able to respond in their mother tongue. In order to increase the validity of the questionnaire in later research, it might be suggested that PA be assessed through the use of a device to measure activity. Also, the results of our study may be a guide in developing new self-reporting scales and in the psychometric examination of other PA instruments. 

## Acknowledgments

This study was supported by the Marmara University Scientific Research Projects Coordination Unit (project number: SAG-C-DRP-171209-0335).
